# To the Editor of the Illinois and Indiana Medical & Surgical Journal

**Published:** 1846-12

**Authors:** John Cooke

**Affiliations:** Manchester, Vt.


### ARTICLE IV.

**To the Editor of the Illinois and Indiana Medical & Surgical Journal:---**

Dear Sir,—A case of some interest recently fell under my observation.

Mr. J. W——— of this town, æt. 30, of a slender constitution and strictly of a scrofulous diathesis, called on me some five years since, with a general derangement of the digestive organs. The constitution thus delicate, I advised him to the moderate use of the blue pill with the extract of conium, and a weak infusion of columbo, which had a favorable effect, and he gradually improved, and was quite well for two or three years, when he again called on me for the same prescription. He then informed me that he had a disease of one of his testicles, and that the same existed when I first saw him, and that it disappeared on following the prescription I gave him, and he supposed the same effect would be produced should he enter again upon the same course. I consequently gave him the same prescription, which he followed some weeks, but to no effect. After passing three or four months, without any medication, he called on me to examine his case, and on examination, I found the left testicle as large as a goose egg, and scirrhus. I advised its extirpation, and in the course of two months he came to the conclusion to have the operation performed, I removed the testicle in March, '45, which weighed 14 oz. He immediately recovered and was able to go out in two weeks after the operation.

His general health improved, and he was able to perform some labor during the summer and autumn following but late in December, '45, his health began to fail, and as the family had become attached to the Thompsonian practice I did not see him until June, '46, when I made him a friendly visit.

At this time, I found him low and feeble, suffering much pain at times from a tumor presenting itself a little to the left and near the lower part of the ensiform cartilege of the sternum. He informed me that the tumor made its appearance some two months previous to my visit. I examined the abdomen and found quite a large tumor occupying nearly the whole of the left hypochondriac region, it was hard and not very tender on pressure. The pain was acute and lancinating. The digestive organs were much impaired, at times very costive, and again attended with a diarrhœa which produced a general degree of prostration.

I saw him again on or about the middle of August, the tumor had increased much in size, occupying the whole of the left hypochondriac and most of the epigastric regions, descending as low in the abdomen as the umbilicus, producing much pressure upon the stomach, heart and lungs, especially when in a horizontal position. There were many elevations and depressions to be distinctly felt at this time, and a degree of elasticity in the whole tumor. I saw him no more until after his death, which took place on the morning of the 8th of September, at which time, myself, with Dr. Tuttle, of this place, were called to make a post mortem examination, and the following phenomena presented itself. On opening the abdomen a large fungoid tumor was to be seen, filling the whole of the left hypochondriac and epigastric regions, and extending as low as the umbilicus, and fluctuation distinctly felt in all parts of tumor, except on the left side, where it seemed more hard and unyielding on pressure. Adhesions to the peritonium had taken place over most of the surface of the tumor, coming in contact with that membrane and required the scalpel to separate them. The tumor involved the duodenum, jejunum and the arch of the colon, which extended through a portion of the tumor and it became necessary to divide them in order to remove it. It also involved the aorta and vena-cava ascendens which much impeded the circulation, and had attached itself to the left kidney which also participated in the disease. It was firmly attached to the spine for six or eight inches in extent. The tumor weighed eight pounds, and presented a great number of abscesses containing from one gill to one drop of pus and resembling the medullary part of the brain in its consistency and oily nature and of a variegated reddish color, in some parts approaching to white.

All other parts, were healthy save a slight enlargement of the liver and three or four small abscesses on its surface.

JOHN COOKE, M. D.

Manchester, Vt., September 11, 1846.

